# The Necessity to Provide an Informed Choice Regarding Venous Thromboembolism Prophylaxis: A Single-Centre Study Observing Clinician Adherence and Opinions on Venous Thromboembolism Prophylaxis

**DOI:** 10.7759/cureus.97154

**Published:** 2025-11-18

**Authors:** Uwais Patel, Ibadur R. A Siddiqui, Azed Lodi, Georges Ziade, Imran Aziz

**Affiliations:** 1 Foundation School, Royal Albert Edward Infirmary, Wrightington, Wigan and Leigh Teaching Hospitals NHS Foundation Trust, Wigan, GBR; 2 Foundation School, St. George's University Hospitals NHS Foundation Trust, London, GBR; 3 Foundation School, Horton General Hospital, Oxford University Hospitals NHS Foundation Trust, Banbury, GBR; 4 Respiratory Medicine, Wrightington, Wigan and Leigh Teaching Hospitals NHS Foundation Trust, Wigan, GBR

**Keywords:** fondaparinux, health inequality, low molecular weight heparin (lmwh), medical ethics, modern medical practice and patient informed choice

## Abstract

Introduction

Venous thromboembolism (VTE) prophylaxis is a critical patient safety measure used in hospitals to prevent venous thromboembolic events. These are often done via nonpharmacological methods, such as anti-embolic (TED™) stockings, or with pharmacological methods, such as low molecular weight heparin (LMWH), which consists of medications such as dalteparin. The synthesis of said medications is derived from bovine or ovine products, thus posing ethical and cultural concerns for those with dietary or religious restrictions. Fondaparinux sodium, a fully synthetic alternative, offers a non-animal-derived option, yet its use remains limited.

Aims

The aim of this study is to explore if those who express a preference for non-animal-derived products are appropriately prescribed fondaparinux, and to educate clinicians on the animal-derived LMWHs with the ability to provide suitable alternatives.

Methodology

A retrospective cohort study was conducted over a seven-week period at Royal Albert Edward Infirmary, Wigan, United Kingdom, from 16/05/25 to 07/07/25. Data were collected from patients who had expressed dietary or religious restrictions regarding animal-derived products and who were prescribed VTE prophylaxis. A secondary survey assessed prescribing clinicians’ knowledge of LMWH derivatives and fondaparinux as an alternative.

Results

Of the 27 newly admitted patients who expressed a preference for non-animal-derived products over the seven-week period, 23 (85%) of these received VTE prophylaxis in the form of LMWH, with four (15%) receiving fondaparinux as a suitable alternative.

Amongst the 24 prescribers surveyed, 12 (50%) knew LMWHs were animal-derived, with 14 prescribers (58.3%) aware of fondaparinux as a synthetic alternative. Despite this, 21 (87.5%) agreed that patients should be informed if medications contain animal-derived ingredients, with 22 (91.7%) supporting informed patient choice.

Conclusion

The use of VTE in a hospital setting is an imperative safety strategy and is used amongst a plethora of specialities. Those patients with expressed preferences for non-animal-derived products had unfortunately received the first-line LMWH prophylaxis, which can be due to either clinicians not having the knowledge that LMWH has animal derivatives, or that there is a suitable alternative to LMWHs. This demonstrates a clear health inequality. Educational initiatives and institutional protocols are recommended to promote patient-centred care and ensure ethical prescribing consistent with individual values.

## Introduction

Thromboprophylaxis is often described as the most important safety strategy in patients admitted to the hospital. This is due to its role in preventing deep vein thrombosis and pulmonary embolism. Pulmonary emboli are commonly cited as the most common cause of preventable in-hospital deaths [1,2]. Due to the prevalence of venous thromboembolism (VTE), current National Institute for Health and Care Excellence (NICE) guidance recommends that all medical patients have their risk factors assessed as soon as possible following admission to the hospital [2].

Thromboprophylaxis can be offered in one of two general methods: mechanical and pharmacological prophylaxis. Mechanical prophylaxis consists of early mobilisation of the patient, anti-embolism (TED™) stockings, and sequential compression devices [3]. For acutely unwell medical patients, following risk assessment, if the risk is viewed to be substantive and too great for mechanical prophylaxis, pharmacological prophylaxis will be required. Recommended pharmacological options, which are currently licensed for adult use, are low molecular weight heparin (LMWH), low-dose unfractionated heparin, and fondaparinux sodium [2-4]. Pharmacological prophylaxis, whilst reducing VTE risk greatly, is associated with an increased bleeding risk and risk specific to the type of anticoagulant used [2-4].

Further to this, NICE guidance recommends that for acutely ill medical patients, in addition to a VTE risk assessment, a bleeding risk assessment must be completed as soon as possible following admission. On balance, if there is a greater risk of VTE and a low risk of bleeding, pharmacological prophylaxis should be offered. For acutely unwell medical patients, LMWH is considered first-line treatment, with fondaparinux sodium considered second-line. It should be noted that there are specific population considerations that affect the choice of pharmacological agent used; for example, fondaparinux sodium is already considered part of the acute coronary syndrome protocol and therefore would be the anticoagulant of choice [1,2].

LMWHs, such as dalteparin and enoxaparin, are fragments of unfractionated heparin. They inhibit coagulation by activating antithrombin III, which binds to and inhibits factor Xa, preventing activation of the final common coagulation pathway [5]. A rare but significant adverse effect of heparins and heparin-derived products such as LMWHs is heparin-induced thrombocytopenia (HIT). HIT is an immune-mediated complication of heparin exposure, presenting clinically as thrombocytopenia with or without thrombosis [6].​

LMWHs are prepared through the controlled depolymerisation of pharmaceutical-grade unfractionated heparin [7,8]. Unfractionated heparin is an animal tissue extract, which traditionally has been porcine-based, although there have been recent developments to derive these products from bovine and ovine sources [8]. This is particularly important as it conflicts with dietary and spiritual requirements of patients, such as veganism, vegetarianism, Islam, and Hinduism, and alternatives in these patient groups should be offered. One such alternative is fondaparinux sodium. Fondaparinux is a synthetic heparin-based anticoagulant, and it does not involve any animal-based products. The process of synthesising fondaparinux is both complex and expensive, which is a key factor in why LMWHs are currently preferred [9].

Fondaparinux sodium is a synthetic pentasaccharide factor Xa inhibitor. It acts by binding to antithrombin III, greatly enhancing antithrombin III’s factor Xa inhibition rate [9,10]. Due to this, fondaparinux is considered a selective factor Xa inhibitor. Fondaparinux is seen to have a longer half-life and duration of action when compared to LMWHs. Moreover, due to its selective inhibition of factor Xa, it has a greater and more predictable anti-factor Xa response [10]. Fondaparinux demonstrates a greatly reduced, but non-zero, risk of HIT [10,11]. Fondaparinux has also been suggested as a treatment for HIT and has been used off-label successfully in the treatment of HIT [11,12].

Following large-scale systemic reviews with meta-analysis, fondaparinux has been demonstrated to have lower rates of VTE for thromboprophylaxis for medical and perioperative patients [13-15]. However, these lower rates of VTE are not associated with a significant difference between LMWH and fondaparinux in reducing symptomatic VTE and all-cause mortality. Furthermore, fondaparinux has been associated with a greater risk of major bleeding, especially post surgery [13-15]. Nonetheless, fondaparinux demonstrates a net clinical benefit over LMWH [13].

The General Medical Council (GMC) promotes a patient-centred approach, according to its professional standards. Doctors are required to consider treatment options that align closely with patients’ needs, preferences, values, and priorities [16]. Since LMWHs are currently cited as first-line agents for VTE prophylaxis, there exists a barrier for doctors to consider patients with dietary, religious, or ethical objections to animal-derived products.

This study was designed as a single-centre retrospective cohort study to review the current compliance of prescribing a LMWH alternative like fondaparinux for VTE prophylaxis to patients who cite a preference for non-animal-derived products. The secondary aim was to observe clinicians' views regarding prescribing VTE prophylaxis in accordance with patient beliefs.

## Materials and methods

Study design and setting

This study was a retrospective study conducted at the Royal Albert Edward Infirmary, Wigan, United Kingdom. The study was conducted for a seven-week period between 16/05/2025 and 07/07/2025.

Ethical guidance

Data collection involved retrospective analysis of routine clinical data. Under UK Health Research Authority guidance, Research Ethics Committee review was not required as no identifiable patient information was reported.

Eligibility

Inclusion criteria included all patients with dietary, personal, or religious constraints preventing the consumption of animal-derived products who were admitted to the Royal Albert Edward Infirmary across all wards and prescribed VTE prophylaxis, and who were greater than 18 years of age between 16/05/2025 and 07/07/2025.

The exclusion criteria included patients who were prescribed treatment dose anticoagulation, diagnosed with coeliac disease, not prescribed VTE prophylaxis, under 18 years of age, and had specific medical requirements requiring LMWH to be prescribed in place of a non-animal-derived alternative.

Data collection

Two datasets were collected, one from patients and one from prescribers. The initial dataset collected data from all patients admitted to the Royal Albert Edward Infirmary. Data collected from patients above 18 years of age were retrospective and included their prescribed medication and any restrictions on the use of animal-derived products.

The second dataset was collected following a four-part self-administered questionnaire centred around clinician knowledge and attitudes on VTE prophylaxis. This was sent to all doctors and prescribers at the Royal Albert Edward Infirmary. The questionnaire is presented in the Appendix.

Outcomes

Two primary outcomes were assessed. The first was to see the percentage of patients who expressed a preference for non-animal-derived VTE prophylaxis who were correctly prescribed an alternative to the animal-based LMWH when prescribed VTE prophylaxis. The second was to observe clinicians' views regarding prescribing VTE prophylaxis in accordance with patient beliefs, and to review current clinician knowledge with regard to non-animal-derived alternatives to LMWH.

## Results

Compliance with prescribing a suitable alternative for patients citing a preference for non-animal-based products

In total, there were 27 newly admitted patients who expressed a preference for non-animal-derived VTE prophylaxis. Of these, 23 were prescribed anticoagulation in the form of LMWH, while five did not ultimately receive anticoagulation because they were discharged prior to medication administration or due to nursing errors. Importantly, among those prescribed prophylaxis, four patients were prescribed fondaparinux as a suitable non-animal-derived alternative, in accordance with their stated preferences (Table 1).

**Table 1:** VTE prophylaxis prescribed to admitted patients citing a preference for non-animal-derived products. VTE: venous thromboembolism; LMWH: low molecular weight heparin.

What patients were prescribed?	Number of patients	Percentage of patients (%)
Prescribed LMWH	23	85
Prescribed and administered LMWH	18	67
Prescribed but not administered LMWH	5	18
Prescribed fondaparinux as an alternative	4	15
Total	27	100

Out of the 27 patients who cited a preference for non-animal-derived VTE prophylaxis, the majority (70.4%) were of Black, Asian, and Minority Ethnic (BAME) backgrounds. With Asian and Asian British people representing the greatest proportion of patients, at 51.9%. The ages of the patients who cited a preference for non-animal-derived VTE prophylaxis ranged between 20 and 94 years, with the two most common age groups being 40-59 and 60-79 years. Furthermore, there was a similar distribution between male and female patients, with 51.85% of those preferring non-animal-derived prophylaxis being male, and 48.15% being female. The most common reason for preferring a non-animal-derived alternative was religious (37.04%); however, dietary (29.63%) and a general avoidance of animal products (33.33%) also represented significant proportions of the population (Table 2).

**Table 2:** Demographic data of patients who cited a preference for non-animal-derived venous thromboembolism prophylaxis.

Characteristic	Frequency (proportion of population, %)
Age group (range = 20-94)	
20-39	6 (22.2)
40-59	8 (29.63)
60-79	8 (29.63)
80-99	5 (18.52)
Sex	
Male	14 (51.85)
Female	13 (48.15)
Ethnicity	
White British	8 (29.6)
Asian/Asian British	14 (51.9)
Black/Black British	1 (3.7)
Other minority background	4 (14.8)
Reason for preference of non-animal alternative	
Avoids animal-derived products	9 (33.33)
Dietary beliefs	8 (29.63)
Religious beliefs	10 (37.04)

Prescribers’ views on enabling appropriate alternatives for patients who cite a preference for non-animal-based products

In total, 24 prescribing clinicians were questioned. Out of these, 87.5% believed that patients should be informed if they are prescribed animal-derived products. Moreover, 50% of clinicians knew that LMWHs are animal-derived, and 58.3% knew that fondaparinux exists as a synthetic alternative in the context of VTE prophylaxis. Finally, 91.7% believed that patients should be allowed to make an informed choice regarding VTE prophylaxis.

## Discussion

There was a clear demonstration that patients who cited a preference for non-animal-derived products were consistently ignored and prescribed animal-derived VTE prophylaxis, i.e., prescribed LMWHs, indicating a clear health inequality for patients who cited a preference for non-animal-derived anticoagulation. Compounded by how the majority of the patients who cited a preference for non-animal-derived prophylaxis are of BAME backgrounds, there is a clear inequality for patients who are of BAME backgrounds. This is not only concerning, but is a continued issue within medicine, with patients who are of minority backgrounds often experiencing an inequality in hospital services [17]. Furthermore, referring to the age groups of those who cited a preference for non-animal-derived alternatives, this is an issue that spans across different ages in a normally distributed manner. Suggesting that this inequality is present across different generations, this is consistent with pre-existing literature, which suggests ethnicity-related health inequalities persist across generations [18]. Suggestions for why inequalities persist in minority ethnic groups are often linked to difficulties in communication for patients who have different native tongues and different cultural norms [17].

Clinicians overwhelmingly agreed that patients should be informed if their prescribed medication is animal-derived, so patients can make an informed choice. This is a position which is echoed in prior literature [19,20]. It is also a sentiment that is relayed in GMC guidance for clinicians [16].

A significant proportion of the clinicians questioned also did not know that LMWHs were animal-derived and that fondaparinux could be used as a suitable alternative for patients who did not want animal-derived products. Considering this and the clear inequality of patients being inappropriately prescribed animal-derived products, it is evident that knowledge gaps could be leading to patients being inappropriately prescribed animal-derived products. This too is an already documented issue, with several previous studies suggesting that one of the primary reasons for this health inequality is a lack of clinician knowledge [19,21].

It is therefore evident that strategies must be employed to strengthen clinicians' knowledge bases. Strategies that could be employed are teaching sessions for prescribing clinicians and, on a national scale, promoting synthetic alternatives like fondaparinux as a suitable alternative. Single-centre studies have shown that repeated teaching cycles and prompt interventions have been successful in reducing the rate at which LMWH has been prescribed over a more suitable alternative [21]. This study is novel in the suggestion that by reducing the number of those who are prescribed LMWH in place of a suitable alternative, an inequality in the standard of in-hospital care for minority ethnic backgrounds could be addressed.

Despite a significant proportion of clinicians recognising that LMWH was animal-derived and knowing that fondaparinux exists as a synthetic alternative, patients were still overwhelmingly prescribed inappropriate VTE prophylaxis. This could likely be a result of clinicians not knowing that their patient has a preference for non-animal-derived products. Due to this, a potential intervention could be asking patients at the front door if they had a preference for non-animal-derived products.

An ethical argument which can be made is that religious governing bodies can provide rulings and exemptions for medication, reducing the number of people who would consider the importance of how their medication was produced [19]. Whilst this is a considerable argument, individual choice is paramount. Therefore, rather than relying on general religious rulings, it is essential to engage each patient in an individual discussion to understand their personal beliefs and preferences. This approach aligns with GMC guidance on consent and shared decision-making, upholding patient autonomy, and promoting culturally sensitive, patient-centred care within the NHS [16].

Limitations of this study include the small sample size and lack of data on recognising patients' native or most familiar language. Despite this, there is a clear understanding that patients who cite an alternative to LMWH have been neglected, and this could be linked to their ethnic backgrounds. Furthermore, this study could be expanded upon by revisiting the economic sustainability of practice, which supports patient-led decision-making with regard to VTE prophylaxis.

## Conclusions

VTE prophylaxis is an important and frequently used safety strategy for patients in hospitals across medical and surgical specialities. Traditionally, VTE prophylaxis can be achieved through either mechanical or pharmacological prophylaxis. Current guidance suggests LMWHs should be considered first-line treatment for pharmacological prophylaxis. This creates an ethical dispute as LMWHs are animal-derived, which would be inconsistent with the lifestyle choices of many patients. A synthetic alternative, in fondaparinux, is available.

This single-centre retrospective study demonstrates that there is a clear health inequality for patients who cite a preference for non-animal-derived products. A significant proportion of prescribing clinicians also did not know that LMWHs were animal-derived and that fondaparinux was a suitable alternative. As such, this health inequality could be a result of a lack of clinician knowledge and difficulties in communication, and strategies should be employed to improve these knowledge gaps and support stronger communication channels.

## Appendices

Questionnaire

Questions asked as part of a four-part questionnaire to prescribing clinicians (Table 3). All were yes or no answers.

**Table 3:** Four-part questionnaire forwarded to prescribing clinicians. LMWHs: low molecular weight heparins; VTE: venous thromboembolism.

Question	Option
Do you think that patients should know if the medication they are taking is animal-derived or not?	Yes or No
Did you know that LMWHs (such as dalteparin and enoxaparin; brand names Fragmin and Arovi) contain porcine-derived products?	Yes or No
Did you know synthetic pentasaccharide factor Xa inhibitors (chemically similar to LMWHs), such as fondaparinux, do not contain any porcine or animal-derived products?	Yes or No
Do you think patients should be allowed to make an informed choice regarding which type of VTE prophylaxis they can take?	Yes or No
